# Quantitative modeling of perovskite-based direct X-ray flat panel detectors

**DOI:** 10.1007/s12200-024-00136-0

**Published:** 2024-09-26

**Authors:** Zihao Song, Gaozhu Wang, Jincong Pang, Zhiping Zheng, Ling Xu, Ying Zhou, Guangda Niu, Jiang Tang

**Affiliations:** 1 Wuhan National Laboratory for Optoelectronics, Huazhong University of Science and Technology, Wuhan, 430074 China; 2 Optical Valley Laboratory, Wuhan, 430074 China; 3 School of Optical and Electronic Information, Huazhong University of Science and Technology, Wuhan, 430074 China

**Keywords:** DQE, X-ray, Detector, Perovskite

## Abstract

**Abstract:**

Direct X-ray detectors based on semiconductors have drawn great attention from researchers in the pursuing of higher imaging quality. However, many previous works focused on the optimization of detection performances but seldomly watch them in an overall view and analyze how they will influence the detective quantum efficiency (DQE) value. Here, we propose a numerical model which shows the quantitative relationship between DQE and the properties of X-ray detectors and electric circuits. Our results point out that pursuing high sensitivity only is meaningless. To reduce the medical X-ray dose by 80%, the requirement for X-ray sensitivity is only at a magnitude of 10^3^ μCGy^−1^⋅cm^−2^. To achieve the DQE = 0.7 at X-ray sensitivity air from 1248 to 8171 μCGy^−1^_air_⋅cm^−2^, the requirements on dark current density ranges from 10 to 100 nA⋅cm^−2^ and the fluctuation of current density should fall in 0.21 to 1.37 nA⋅cm^−2^.

**Graphical Abstract:**

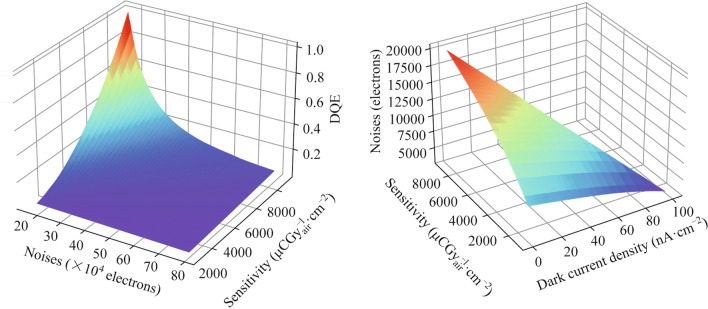

## Introduction

X-ray flat-panel detectors are widely applied in medical imaging and nondestructive testing [1]. The detective quantum efficiency (DQE), which is used to evaluate their imaging performance, depends on the sensitivity, spatial resolution and uniformity of detectors [2]. At present, the market is dominated by indirect detectors based on scintillators like thallium-doped CsI, the DQE for most of them is under 0.7 [3]. The Achilles’ heel of indirect detectors is the strong optical crosstalk and the low sensitivity (< 10^3^ μCGy^−1^_air_⋅cm^−2^) from the X-ray – visible light—electron conversion process [4]. While, the direct detectors based on semiconductors can convert X-ray to electrons in one step, which gives them higher spatial resolution and sensitivity, thus higher DQE theoretically. As a result, researchers put great effort into studying direct detectors like perovskite-based detectors, including a-Se, CdTe and perovskite based detectors [5, 6].

Perovskites-based X-ray detectors (PeroXD) have shown high X-ray sensitivities (> 10^4^ μCGy^−1^_air_⋅cm^−2^) and low detection limits [7–9], which proves that they are great candidates for next-generation X-ray detectors. Many previous works focused on the optimization of the crystal quality and material composition to improve the sensitivity and detection limit performance [10, 11]. For further application, perovskites should be integrated with the front-end pixel circuits such as thin film transistors (TFT) [1, 4, 12]. However, previous studies have paid little attention to the overall performance of the flat panel detectors, i.e., the key factors affecting the DQE value. Simply pursuing high sensitivity is meaningless to the DQE value. Moreover, previous studies on theoretical modeling of DQE seldomly mentioned the relationship with the direct X-ray detectors, thus unable to guide the perovskite flat panel detectors toward further applications.

This work focuses on building the quantitative relationship between DQE and the related properties of perovskite detectors like sensitivity, dark current density and uniformity. Our method includes decomposing the system-level parameters in the DQE definition equation (conversion gain, image noises, etc.) into device-level parameters (X-ray sensitivity, dark current, electron noises, etc.). We also calculated the quantitative values of detector-level parameters at high DQE under the restriction of circuit properties (full-well capacity, frame rate, etc.). We find out that perovskites with sensitivities at the level of 10^3^ μCGy^−1^_air_⋅cm^−2^ is enough to reduce the imaging dose of general radiography by 80% and achieving high DQE of 0.7 requires dark current density from 10 to 100 nA⋅cm^−2^ and the fluctuation of current density from 0.21 to 1.37 nA⋅cm^−2^ for sensitivities from 1248 to 8171 μCGy^−1^_air_⋅cm^−2^.

## Results and discussion

### First principle decomposing of DQE

According to IEC-62220–1 [2], the DQE is defined as:

1 $$\begin{array}{c}\text{DQE}\left(f\right)={G}^{2}{\text{MTF}}^{2}\left(f\right)\frac{{\text{NPS}}_{\text{in}}}{{\text{NPS}}_{\text{out}}\left(f\right)} \end{array},$$

where *G* is the conversion gain of the X-ray detector, MTF is the modulation transfer function, NPS_in_ and NPS_out_ represent the noise power spectrum (NPS) of the input and output signals respectively. The spatial frequency of signals is denoted with *f*.

2 $$\begin{array}{c}\begin{array}{c}G=\frac{\text{Sensitivity}}{e\cdot Q}\end{array}\end{array}.$$

The conversion gain is defined as the number of electrons generated per incident X-ray photon, which essentially represents the same thing as the X-ray sensitivity. The X-ray sensitivity is defined as the amount of charge generated per unit of X-ray dose per unit area. According to their definition, the relationship between the conversion gain and the X-ray sensitivity of detectors can be defined by Eq. (2), where *e* is the elementary charge and *Q* represents the X-ray fluence of different spectra [2]. For RQA5 spectrum used in general radiography,* Q* = 30174 mm^−2^⋅μGy^−1^.

3 $$\begin{array}{c}\text{DQE}={\text{MTF}}^{2}\left(f\right){\left(\frac{\text{Sensitivity}}{e\cdot Q}\right)}^{2}\frac{{\text{NPS}}_{\text{in}}}{{\text{NPS}}_{\text{out}}}\\\end{array}.$$

We can derive the relationship between DQE and X-ray sensitivity (Eq. (3)) from Eqs. (1) and (2).

4 $$\begin{array}{c}{\text{NPS}}_{\text{in}}=\text{Dose}\cdot Q\cdot A\\\end{array}.$$

The relationship between the noise power spectrum and the fluence of the X-ray spectrum is shown in Eq. (4), where *A* is the area of pixel. The parameter *A* is introduced to NPS_in_ to remove its dependence of the area, because the NPS_out_ is expressed in electrons which is area independent as shown in Eq. (5).

5 $$\begin{array}{c}{\text{NPS}}_{\text{out}}={\text{Noise}}_{\text{tot}}^{2}=\sum_{i}^{n}{\text{Noise}}_{i}^{2}\\\end{array}.$$

The value of NPS_out_ is the square of the amplitude of noise. The total noise is the quadratic sum of noises from different sources (Eq. (5)).

6 $$\begin{array}{c}\text{DQE}={\text{MTF}}^{2}\left(f\right){\left(\frac{\text{Sensitivity}}{e\cdot Q}\right)}^{2}\frac{\text{Dose}\cdot Q\cdot A}{\sum_{i}^{n}{\text{Noise}}_{i}^{2}}\\\end{array}.$$

Equation (6) shows the relation between DQE, X-ray sensitivity, and electric noise of X-ray detectors.

7 $$\begin{array}{c}\begin{array}{c}\frac{\text{Sensitivity}\cdot \text{Dose}\cdot A+\frac{{J}_{\text{dark}}\cdot A}{\text{fps}}}{e}={N}_{\text{max}}-\text{Noise}\approx {N}_{\text{max}}\end{array}\\\end{array}.$$

The total signal includes dark signal, X-ray induced signal and noise signal. It should be less than the full-well capacity of electric circuits as shown in Eq. (7), in which *J*_dark_ represents dark current density, fps represents the frame rate of images, and ${N}_{\text{max}}$ represents the full-well capacity of electric circuits.

8 $$\begin{array}{c}\begin{array}{c}\text{DQE}\left(0\right)=\left({N}_{\text{max}}-\frac{{J}_{\text{dark}}\cdot A}{\text{fps}\cdot e}\right)\cdot \frac{\text{Sensitivity}}{{\text{Noise}}_{\text{tot}}^{2}\cdot e\cdot Q}\end{array}\\\end{array}.$$

The ultimate relationship between DQE(0), X-ray sensitivity, dark current density and electric noise of X-ray detectors (Eq. (8)) can be derived from Eqs. (6) and (7), where MTF(0) equals 1.

### Calculation of quantitative requirements on detectors for low-dose X-ray imaging

In this section, the quantitative requirements for the electronic circuit of Dexela 2923 are calculated as a demonstration on the application of our model. The Dexela 2923 is a commercial X-ray image sensor of PerkinElmer Inc. for multiple non-destructive testing applications.

The technical details of Dexela 2923 are shown in Table 1. The rated dose of Dexela 2923 is 2.5 μGy for general radiography.

**Table 1:** Maximum X-ray sensitivity of Dexela 2923 at different imaging setups

Mode	Capacity (e)	Dose (μGy_air_)	Max. sensitivity (μCGy^−1^_air_⋅cm^−2^)
General radiography	1570000	2.5	1788.5
General radiography	1570000	0.5	8942.5

9 $$\begin{array}{c}{\text{Sensitivity}}_{\text{max}}=\frac{{N}_{\text{max}}\cdot e}{\text{Dose}\cdot A}\\\end{array}.$$

The maximum compatible sensitivity (Sensitivity_max_) of Dexela 2923 can be calculated with Eq. (9), for which the pixel area is 75 μm × 75 μm. We calculated the Sensitivity_max_ of Dexela 2923 at rated dose and 1/5 of rated dose respectively. The result shows that the maximum sensitivity should be no more than 233.5 and 8942.5 μCGy^−1^_air_⋅cm^−2^ for general radiography at 1/5 of rated dose respectively. Otherwise, the X-ray induced signals will be more than the electric circuits can collect. The following analysis is based on 1/5 of rated dose as a demonstration of calculating the requirements of low dose high DQE imaging.

**Fig. 1:**
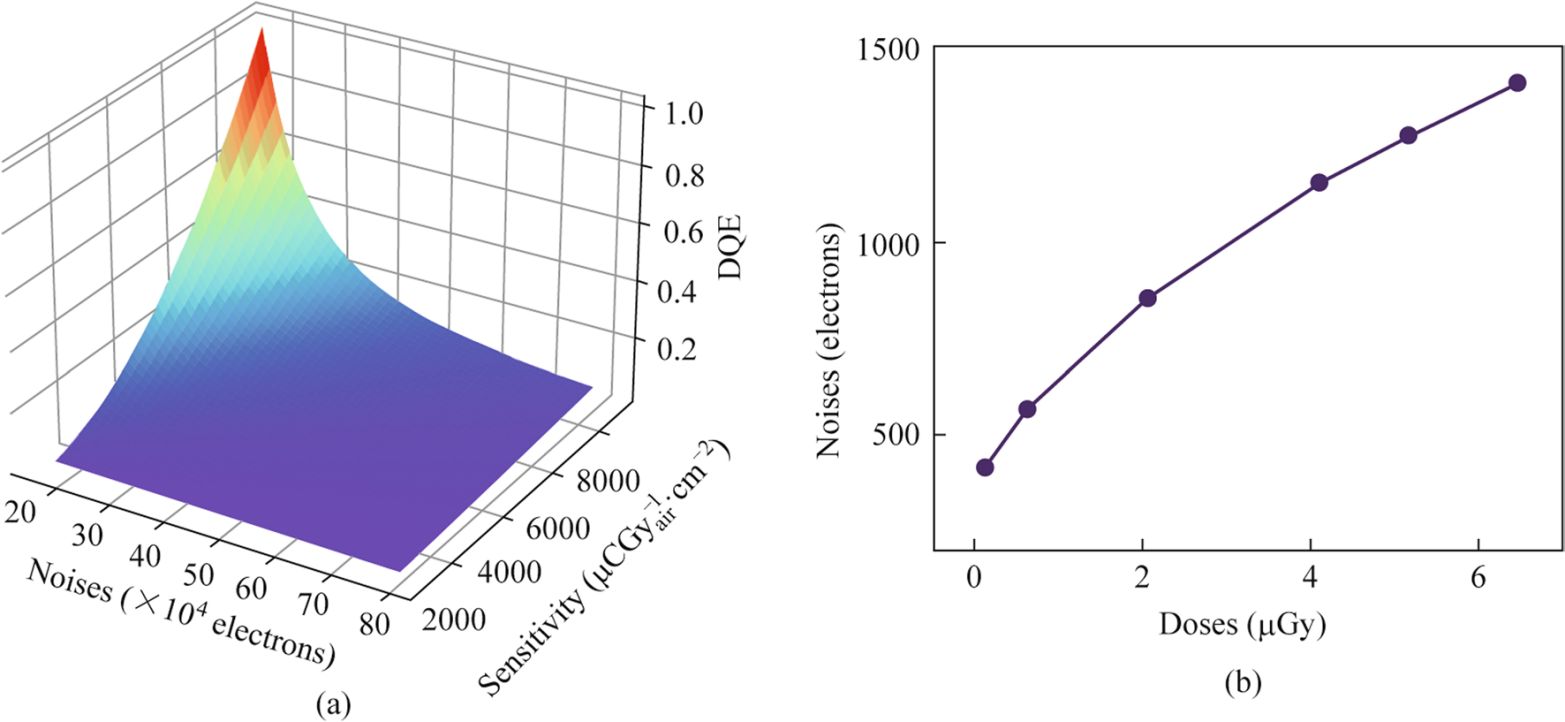
**a** Relationship between noise, sensitivity, and DQE for Dexela 2923 for general radiography at 0.5 μGy. **b** Electronical noise of a pixel for Dexela 2923 for general radiography

The relationship between noise, sensitivity, and DQE for Dexela 2923 for general radiography at 1/5 of normal dose is shown in Fig. 1a, which are calculated using Eq. (6) with *f* = 0 lp⋅mm^−1^. The results shows that lower sensitivity requires lower noise (higher uniformity) to achieve high DQE.

10 $$\begin{array}{c}{\text{Noise}}_{\text{pix}}^{2}=K\cdot \text{Dose}+{\text{Noise}}_{\text{read}}^{2}\\\end{array}.$$

To analyze the major source of noises,the electrical noises of each signal pixel at different doses are calculated using Eq. (10), where Noise_pix_ is the noise of pixel at different X-ray doses. The dose-dependent conversion gain is denoted with *K*, and Noise_read_ is the intrinsic noise of the readout electric circuit.

The experimental *K* of Dexela 2923 is 285,372 electron/μGy_air_ (811.7 μCGy^−1^_air⋅_cm^−2^), far below the Sensitivity_max_. The dose dependent noise of signal pixel is shown in Fig. 1b, which is much lower that the noise shown in Fig. 1a. It indicate that the majority of noises come from the pixel-pixel differences rather than the time dependent fluctuation of each pixels.

**Fig. 2:**
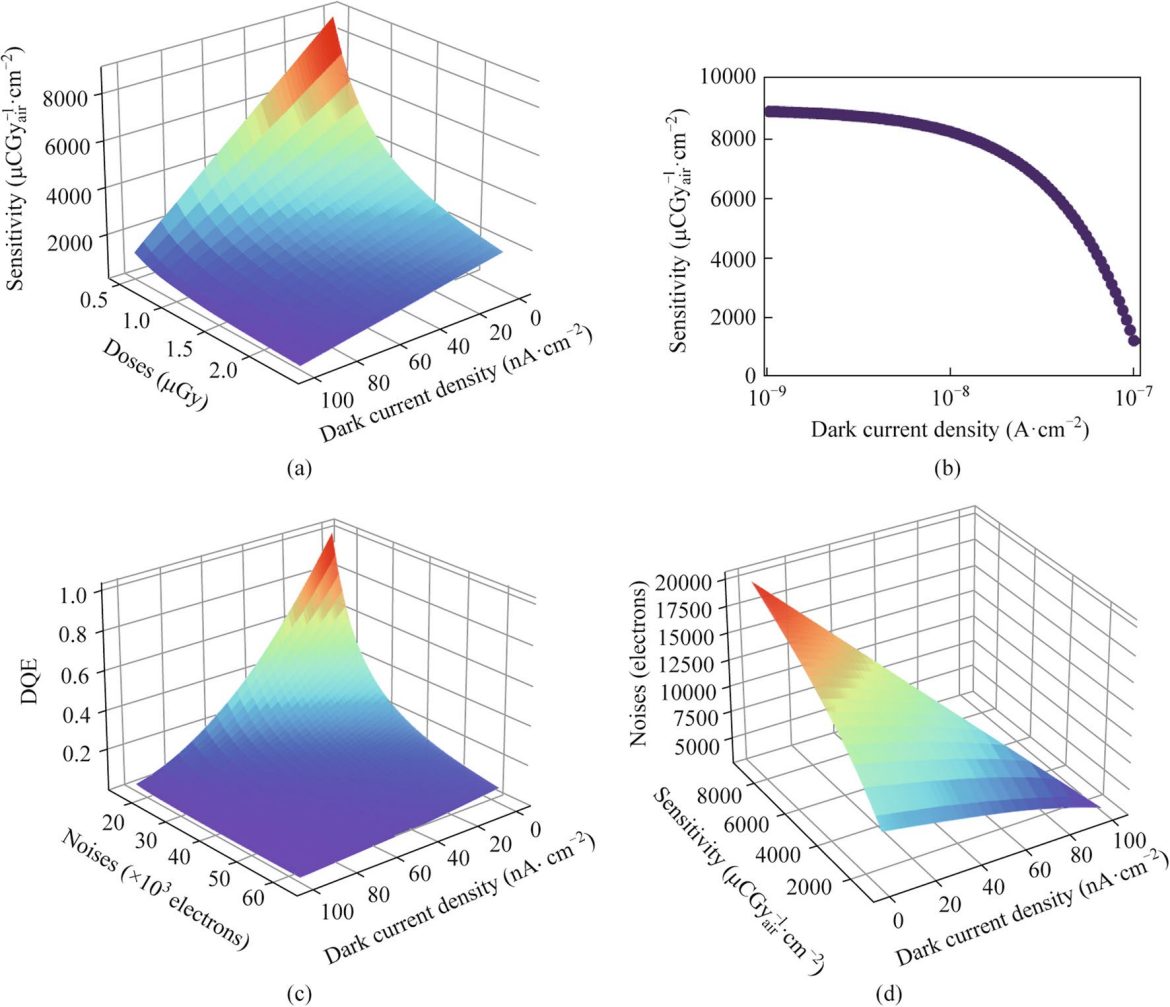
**a** Relation ship between dark current density, full-well dose, and sensitivity for Dexela 2923. **b** Relationship between sensitivity and dark current density for Dexela 2923. **c** DQE for Dexela 2923 at different noises and dark current density when reaching maximum well. **d** Noises required to achieve DQE = 0.7 for different dark current density and Sensitivity at 0.5 µ${\text{Gy}}$ for Dexela 2923

To find out how the full-well capacity of the electric circuits and the dark current will influence the DQE of X-ray detectors, we carry out a numerical simulation with Eqs. (7) and (8), under the assumption that dark signals and X-ray-induced signals are just enough to fill the capacitors of electric circuits. Figure 2a shows the highest X-ray sensitivity that detectors can achieve at different dark current densities and imaging doses. Figure 2b presents the competitive relationship between maximum X-ray sensitivity and dark current density at 0.5 μGy specifically. We can find out that the maximum DQE that X-ray detectors can achieve decreases with the increase of dark current density in Fig. 2c.

The relationship between dark current density, X-ray sensitivity, and total electrical noise at DQE = 0.7 and Dose = 0.5 μGy is shown in Fig. 2d. The results points out that achieving X-ray imaging with DQE = 0.7 at 1/5 of normal medical doses only requires the sensitivity at a level of 10^3^ μCGy^−1^_air_⋅cm^−2^ when dark current density is around 10^−8^ A⋅cm^−2^, which limits the maximum sensitivity that can be achieved. As the dark current density decreases from 100 to 10 nA⋅cm^−2^, the maximum sensitivity increases from around 1248 to 8171 μCGy^−1^_air_⋅cm^−2^. The required uniformity depends on the sensitivities of X-ray detector. In the above situations, when the allowed variance of the signal of different pixels decreases from 18,612 electrons (1.37 nA⋅cm^−2^) to 7610 electrons (0.21 nA⋅cm^−2^), the maximum sensitivity decreases from 8171 to 1248 μCGy^−1^_air_⋅cm^−2^.

## Conclusion

We proposed the quantitative relationship between DQE and the properties of X-ray detectors, which is a helpful tool to tell researchers the requirements that X-ray detectors should meet to achieve high-quality X-ray imaging. We made a demonstration with Dexela 2923 to show how our model can be used and how to analyze what should be improved for perovskite-based detectors. We believe this work provides a deep insight into how the fundamental properties of X-ray detectors will influence imaging quality.

## Data Availability

The data that support the findings of this study are available from the corresponding author, upon reasonable request.
